# Supplementary material for: Clinical Effectiveness of Renal Transplant Outpatient Pharmaceutical Care Services in Korea
Source: Healthcare (Basel). 2023 Sep 21;11(18):2597. doi: 10.3390/healthcare11182597 (PMC10531252; doi:10.3390/healthcare11182597)
Supplement: Supplementary file 1 [file healthcare-11-02597-s001.zip › healthcare-2592594-supplementary.pdf]

Supplementary Table S1. Baseline characteristics for sensitiviy analysis

	Pre-Match			Post-Match		
**Variables**	**Control N= 283**	**PCS N= 239**	**STD**	**Control N= 167**	**PCS N= 167**	**STD**
Sex (male)	173 (61.1)	136 (56.9)	0.06	102 (61.1)	94 (56.3)	0.1
Age (years)	53.8 ± 14.4	54.0 ± 14.0	0.01	53.6 ± 14.5	54.6 ± 13.7	0.07
Diagnosis						
Kidney transplantation	193 (68.2)	205 (85.8)	0.4	141 (84.4)	141 (84.4)	0
Chronic kidney disease	74 (26.2)	60 (25.1)	-0.02	44 (26.4)	45 (27.0)	-0.01
Kidney donor	40 (14.1)	10 (4.2)	-0.4	7 (4.2)	8 (4.8)	-0.02
Hypertension	159 (56.2)	184 (77.0)	0.5	109 (65.3)	121 (72.5)	0.1
Diabetes Mellitus	75 (26.5)	104 (43.5)	0.4	55 (32.9)	67 (40.1)	0.1
Dyslipidemia	176 (68.2)	175 (73.2)	0.2	121 (72.5)	122 (73.1)	0.01
Number of oupatient visits	6.9 ± 3.5	10.4 ± 5.3	0.8			0.07
Number of medications	7.4 ± 7.1	13.3 ± 10.4	0.7			0.1
Medications						
Diuretics	31 (11.0)	71 (29.7)	0.5	24 (14.4)	31 (18.6)	0.1
Potassium-lowering agents	5 (1.8)	14 (5.9)	0.2	5 (3.0)	4 (2.4)	-0.03
Alkalizing agents	11 (3.9)	31 (13.0)	0.3	8 (4.8)	8 (4.8)	0
Antibiotics	82 (29.0)	116 (48.5)	0.4	61 (36.5)	68 (40.7)	0.09
Immunosuppressants	196 (69.3)	205 (85.8)	0.4	141 (84.4)	141 (84.4)	0
CKD_MBD medications	34 (12.0)	59 (24.7)	0.3	25 (15.0)	24 (14.4)	-0.02
Laboratory findings						
Serum creatinine (mg/dL)	1.29 ± 0.7	1.5 ± 1.0	0.3	1.29 ± 0.7	1.3 ± 0.7	0.04
BUN (mg/dL)	19.39 ± 9.5	24.1± 13.1	0.4	20.6 ± 11.3	21.2 ± 9.2	0.06
eGFR(mL/min/1.73m^2)	64.49 ± 21.4	55.9 ± 23.2	-0.4	62.5 ± 21.6	60.3 ± 21.6	-0.1
Calcium (mg/dL)	9.4 ± 0.4	9.3 ± 0.5	-0.2	9.4 ± 0.5	9.3 ± 0.5	-0.06
Cholesterol (mg/dL)	182.1 ± 30.1	181.7 ± 33.5	-0.01	184.0 ± 29.7	182.4 ± 32.8	-0.05
Glucose (mg/dL)	108.1 ± 25.5	113.7 ± 44.0	0.2	111.9 ± 30.1	114.1 ± 43.4	0.06
Hemoglobin (g/dL)	13.6 ± 1.7	13.2± ±0.8	-0.2	13.5 ± 1.8	13.3 ± 1.8	-0.1
Albumin (g/dL)	4.5 ± 0.3	4.3 ± 0.4	-0.3	4.4 ± 0.4	4.4 ± 0.4	-0.08
Total bilirubin (mg/dL)	0.8 ± 0.3	0.8 ± 0.3	0.2	0.8 ± 0.4	0.8 ± 0.3	-0.04
Uric acid (mg/dL)	5.8 ± 1.5	6.1 ± 1.7	0.2	5.8 ± 1.5	5.9 ± 1.6	-0.1
Platelet (10^9/L)	224.2 ± 57.5	221 ± 59.0	-0.06	222.7 ± 60.5	223.2 ± 59.9	0.01
White Blood Cell (10^9/L)	7.0 ± 1.9	7.4 ± 2.5	0.2	7.2 ± 1.9	7.3 ± 2.3	0.07

Supplementary Table S2. Sensitiviy Analysis for Study Outcomes

Outcomes	Incidence or measured change		aOR or *P* value
	Control	PCS	
End Stage Renal Disease	30/167 (17.9)	19/167(11.3)	0.34 (0.14 – 0.78)
Change in estimated glomerular filtration rate between before and after study period.	-6.8 mL/min/1.73m^2	-1.7 mL/min/1.73m^2	0.06

Values are represented as number (%); aOR, adjusted odds ratio; PCS, pharmaceutical care services;
